# Sustained Reduction of the Dengue Vector Population Resulting from an Integrated Control Strategy Applied in Two Brazilian Cities

**DOI:** 10.1371/journal.pone.0067682

**Published:** 2013-07-03

**Authors:** Lêda N. Regis, Ridelane Veiga Acioli, José Constantino Silveira, Maria Alice Varjal Melo-Santos, Wayner Vieira Souza, Cândida M. Nogueira. Ribeiro, Juliana C. Serafim. da Silva, Antonio Miguel Vieira Monteiro, Cláudia M. F. Oliveira, Rosângela M. R. Barbosa, Cynthia Braga, Marco Aurélio Benedetti Rodrigues, Marilú Gomes N. M. Silva, Paulo Justiniano Ribeiro Jr., Wagner Hugo Bonat, Liliam César de Castro Medeiros, Marilia Sa Carvalho, André Freire Furtado

**Affiliations:** 1 Departameto de Entomologia, Fundação Oswaldo Cruz-Fiocruz-Pe, Recife-PE, Brazil; 2 Secretaria Estadual de Saúde, Recife-PE, Brazil; 3 Departameto de Saúde Coletiva, Fundação Oswaldo Cruz-Fiofruz-PE, Recife-PE, Brazil; 4 Secretaria Municipal de Saúde, Santa Cruz do Capibaribe-PE, Brazil; 5 Secretaria Municipal de Saúde, Ipojuca-PE, Brazil; 6 Divisão de Processamento de Imagens, Instituto Nacional de Pesquisas Espaciais-INPE, São José dos Campos-SP, Brazil; 7 Departameto de Parasitologia, Fundação Oswaldo Cruz-Fiocruz-PE, Recife-PE, Brazil; 8 Departameto de Eletrônica e Sistemas, Universidade Federal de Pernambuco, Recife-PE, Brazil; 9 Departameto de Estatística, Universidade Federal do Paraná, Curitiba-PR, Brazil; 10 Centro de Ciência do Sistema Terrestre, Instituto Nacional de Pesquisas Espaciais-INPE, São José dos Campos-SP, Brazil; 11 Fundação Oswaldo Cruz-Fiocruz, Rio de Janeiro-RJ, Brazil; 12 Departameto de Virologia, Fundação Oswaldo Cruz-Fiocruz-PE, Recife-PE, Brazil; Universidade Federal do Rio de Janeiro, Brazil

## Abstract

*Aedes aegypti* has developed evolution-driven adaptations for surviving in the domestic human habitat. Several trap models have been designed considering these strategies and tested for monitoring this efficient vector of Dengue. Here, we report a real-scale evaluation of a system for monitoring and controlling mosquito populations based on egg sampling coupled with geographic information systems technology. The SMCP-Aedes, a system based on open technology and open data standards, was set up from March/2008 to October/2011 as a pilot trial in two sites of Pernambuco -Brazil: Ipojuca (10,000 residents) and Santa Cruz (83,000), in a joint effort of health authorities and staff, and a network of scientists providing scientific support. A widespread infestation by Aedes was found in both sites in 2008–2009, with 96.8%–100% trap positivity. Egg densities were markedly higher in SCC than in Ipojuca. A 90% decrease in egg density was recorded in SCC after two years of sustained control pressure imposed by suppression of >7,500,000 eggs and >3,200 adults, plus larval control by adding fishes to cisterns. In Ipojuca, 1.1 million mosquito eggs were suppressed and a 77% reduction in egg density was achieved. This study aimed at assessing the applicability of a system using GIS and spatial statistic analysis tools for quantitative assessment of mosquito populations. It also provided useful information on the requirements for reducing well-established mosquito populations. Results from two cities led us to conclude that the success in markedly reducing an Aedes population required the appropriate choice of control measures for sustained mass elimination guided by a user-friendly mosquito surveillance system. The system was able to support interventional decisions and to assess the program’s success. Additionally, it created a stimulating environment for health staff and residents, which had a positive impact on their commitment to the dengue control program.

## Introduction

*Aedes aegypti* Limnaeus 1762 (Diptera:Culicidae) populations appear to be currently well established in most households at almost every tropical urban setting and are also established in some sub-tropical areas. The main consequence is that two fifths of the world's population is potentially exposed to four infections by the dengue viruses, resulting in 50 million infections annually, as estimated by the World Health Organization. Dengue fever is currently considered as one of the fastest spreading diseases in the world [1]. Even if an efficient dengue fever vaccine becomes available, *A. aegypti and A. albopictus* mosquitoes established in urban environments will remain as a matter of concern as these Culicids are highly efficient vectors of other arbovirusis such as, Yellow Fever, West Nile viruses and Chikungunya. For Brazil and other South American countries, widespread infestation by *A. aegypti* is a relatively recent scenario: a few decades ago villages and cities were considered free of dengue vectors. Rapid spread and stable installation of the main DENV vector *A. aegypti* in urban and semi-urban territories are greatly favored by evolution-driven adaptations to the human host [2] and to unstable aquatic habitats commonly found in the human house. Some of these adaptations are the mosquito’s ability to colonize a very wide variety of water holding containers, to spread eggs from the same batch on different sites, and the high resistance of the egg chorion. Due to these biological characteristics, and to the high number of diverse objects that hold water often found in modern urban environments, the classical method of visual inspection to detect larvae/pupae is extremely difficult and time-consuming, resulting in an ineffective way to monitor urban mosquito populations. Moreover, it is unlikely that classical control interventions based mainly on the application of larvicides in aquatic habitats will succeed on reducing dengue vector populations. Nevertheless, this is still the method used in most dengue endemic countries and despite the large amounts of financial resources spent in vector control for over a decade, most urban territories in those countries remain heavily infested by *Ae. aegypti*, as is the case of Brazil [3].

Several experimental and practical evidences obtained from studies developed in different countries show that the use of traps is a more appropriated strategy to monitor *Ae. aegypti* and *Ae. albopictus* than classical surveillance methods. Different models of ovitraps have been clearly shown as effective monitoring tools when integrated to entomological surveillance systems able to generate quantitative information on mosquito presence and densities [4]–[14]. Such systems are essential for directing control actions and measuring their impact on the vector abundance. A great potential of traps as a mass attract-and-kill strategy for reducing mosquito population has been revealed in some studies [7], [10], [15]–[17], [18]. Applying a sensitive surveillance system (SMCP-*Aedes*) in a district of Recife City, Brazil, we have shown that *Aedes* population boosts resulting from massive eggs hatching promoted by rainfall that follows a dry season, can be prevented through this control strategy by eliminating, at the dry season, more than 6 million eggs collected in 4000 traps throughout 4 months [7].

The SMCP-*Aedes* - Monitoring System and Population Control for urban *Aedes,* is an entomological surveillance framework to provide baseline data for dengue epidemiological surveillance. It consists of an all-integrated approach supported by the intensive use of the web and free software to collect, store, analyze and disseminate information on the spatial-temporal distribution of the estimated density of *Aedes*, based on data that is systematically collected with the use of ovitraps and integrated through the use of open geospatial technologies such as GIS, Remote Sensing Images and Spatial Statistics analytical tools [19]. This system was developed based on a 3-year longitudinal experiment using 460 georeferenced ovtitraps in seven districts of Recife [7]. It was subsequently (2008–2011) settled as a pilot program in two cities and since 2010 it has been set up in an Oceanic Island in Pernambuco, all endemic areas for dengue.

In this article, we report the results of a strategy based on integrated control interventions guided by mosquito surveillance applied by the local health authorities and staff coupled with scientific team support and community participation. This strategy was successfully able to reduce mosquito populations in Santa Cruz do Capibaribe-PE and Ipojuca-PE, Brazil. Our ultimate goals were to evaluate at real-scale and to improve the applicability of an environmentally friend vector monitoring and control system aiming to contribute to reduce dengue virus transmission.

## Methods

### Ethics Statement

This study was reviewed and approved by the Ethics Committee of the CPqAM-Fiocruz-PE, Brazil (No. 14/04). Field works were carried out by the Municipal Health personnel, as part of the Municipal Dengue Control Program in agreement with the Brazilian rules for dengue control in endemic areas. In areas where the presence of *Aedes aegypti* is confirmed, a written consent for house visits and mosquito control interventions by the municipal government is not required. With the consent of the municipal health authorities the scientific team had full access to all data generated by the use of the SMCP-*Aedes*, as well as the teams from the Health Secretary had access to all the results of analyzes of the data made by the scientific team.

The SMCP-*Aedes* was evaluated from March 2008 to October 2011, in a joint effort of the state health authorities, local health staff and a scientific team organized in a network.

### Implementation of the Program

#### 1. Site selection

**Figure 1:**
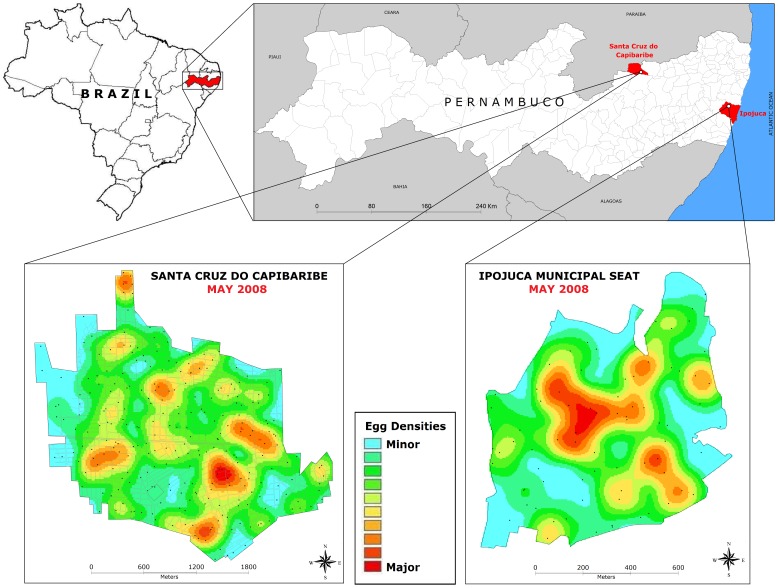
Spatial distribution of mosquito egg for Ipojuca and Santa Cruz do Capibaribe-PE, Brazil, in May 2008. The Kernel maps showing smoothed egg densities are based on the number of eggs deposited in each sentinel-ovitrap during one month. Data from the sentinel network encompassing 262 ovitraps distributed over a 5.6 km^2^ urban area in Santa Cruz do Capibaribe and 75 ovitraps over a 0.8 km^2^ urban area of Ipojuca municipal seat.

The system was evaluated in two municipalities chosen by the State Health Department of Pernambuco based on dengue fever incidence, different geographic characteristics, and commitment level of the health staff: Ipojuca is located on the coast at 50.2 km from Recife, the capital of the state; Santa Cruz do Capibaribe is located in the Semi-Arid region of the state, at 194.3 km from Recife (Figure 1).

Santa Cruz do Capibaribe (7° 57′ 27″ S and 36°12′17′′ W, 438 m above sea level) has annual mean temperature of 26.9°C (min 21° max 32.5° C) and 360.3 mm of annual average rainfall, which occurs from January to July. The municipality, here referred as SCC, has a total area of 335 km^2^ with 87582 inhabitants [20]. From 2001 to 2008 SCC notified 2394 dengue cases (1124 during the Denv-3 epidemics in 2002), with most of them occurring from January to April. The Premise Index (PI, percentage of inspected premises found to have containers positive for *Ae. aegypti* larvae/pupae), used as an indicator of dengue risk by the PNCD-National Program for Dengue Control, ranged from 3.8 to 12.9 in 2007 and 2008, with annual means of 4.8 and 9.4, respectively. The present study was conducted in an urban area of 5.67 km^2^ where 83161 residents live. Access to water is not regular and residents often store water in underground cisterns (approximately 20000 cisterns with an average capacity of 10000 L), which are potential breeding places for culicids. The field work team of the dengue control program was composed of 32 endemic agents (EA), three supervisors and one coordinator. Ipojuca (08°24′00′′ S and 35°03′45′ W) has a population of 80637 inhabitants [20] and a 532 Km^2^ area**.** The annual mean temperature is 26.1°C (min 21.8°C - max 30.7°C), with a 1719.4 mm mean annual rainfall concentrated from March to August. Ipojuca notified 3060 dengue cases (1586 confirmed cases) from 2000 to 2008, with approximately 80% of them occurring in January-June. The PI was 0.8 in 2007 and 0.4 in 2008. The study was conducted in the Municipal Seat, referred as IpojucaMS, an area of 0.8 km^2^ with 10037 people living at. The local field team was composed by eight EA and one supervisor.

For administrative reasons the PNCD procedures for mosquito control, implemented since 1997, were maintained during this study in both study areas. PNCD vector control activities comprise routine bimonthly application of temephos (larvicide); an annual campaign for source elimination - the Dengue Day; and application of organophosporous (OP) or piretroids adulticides through ULV in the case of epidemics. Following a decision by the PNCD, the OP temephos was replaced by chitin synthesis inhibitors in January/2011in IpojucaMS and in May/2011 in SCC.

#### 2. Pre-trial

Prior to implementation, the procedures proposed by the SMCP-*Aedes* were approved by the management and technical staff of the municipal health department, and then discussed in open meetings (April 2008) with the local authorities of Education, Urban Environment Management and Social Actions departments, the Municipal Health Council, and citizens. Training and workshops were offered to the local dengue program staff by the scientific research team in April 2008 and in September 2008, providing theoretical and practical knowledge on the strategies, methods and tools to be adopted by the SMCP-*Aedes* for mosquito monitoring and control.

#### 3. Sentinel network set up

The number of traps for surveying each site was calculated based on a logistic function according to house density, as described in Regis et al (2009). For ovitrap distribution within the study area, grids with 40 m×40 m cells were overlaid in the study area map. The grids were made of approximately 3500 cells in SCC and 490 in IpojucaMS. Out of these, a sample of 262 and 75 cells were systematically and randomly chosen in SCC and IpojucaMS, respectively. The sentinel networks were installed in May 2008 by the field team of each local health service equipped with maps indicating the quadrants for geo-referenced sentinel-ovitraps (S-ovt) location, a GPS, Bti-larvicide and labeled traps. The networks were composed of 262 S-ovt in SCC and 75 in IpojucaSM. Each S-ovt was installed at a fixed sampling station, hung 1 m above ground level outdoor of the residence, in the shade and protected from rainfall. Geographical coordinates of trap location were taken using a Global Positioning System (GPS) and entered into the SMCP-*Aedes* geographical database. This database is based on open technology, open protocols and open data and was designed to make available reports and data analysis [19]. The S-ovt model used for sampling *Aedes* eggs, described in Regis et al [7], consists of a black plastic cup filled with two liters of water, two 5×15 cm wood paddles as egg substrates and two or four drops of a Bti-based product as larvicide, to prevent the trap from becoming a mosquito breeding site [6]. Liquid Bti-products in 30 or 50 ml bottles (Bt-horus, Btheck, Brasilia DF, Brazil or Killarv-Bti, Caruaru-PE, Brazil) were chosen instead of granular formulations, because they are more practical to transport and do not deposit residues on the paddles. Each ovitrap was labeled with an identification code, the set up date and a telephone number. The first household visit was aimed at explaining the project to and obtaining the agreement of the householder, followed by trap installation. Monthly checking of the S-ovt was conducted to replace paddles, water and Bti. The paddles taken each month from Ipojuca (150 units) and SCC (524) were delivered to the Department of Entomology/Fiocruz-PE in Recife for egg counting.

*Semi-automatic egg counting*: A system for acquiring digital images, the SDP-Egg Counting System, was developed to hasten and improve the process of counting the eggs laid on the paddles [21]. The system consists of an equipment to perform optical scanning images of the trap paddles and of software installed on a desktop computer to assist egg counting. Image acquisition starts when the user enters the paddle in the system. *Aedes* eggs are recognized by the operator on the digitalized image. The counting is based on mouse click over the picture and the number of identified eggs is registered and transmitted via web to the SMCP-*Aedes* Geographic Database [19]. Egg counting through the SDP system allowed analysis of paddles to be done around two times faster than the conventional microscopic method.

*Aedes* species identification. Fourth instar larvae (L4) reared from field-collected eggs were used for species identification. Egg samples from at least 70 different sentinel stations were used. For sample size calculation, it was considered relevant to detect *Aedes* species other than *A. aegypti*, with at least 0.1% prevalence. Assuming a hyper-geometric distribution with p = 1/1000, the sample size required per village was set as 1000 identified individuals.

#### 4. Control Interventions

The strategy proposed in the SMCP-*Aedes* combines: i) the diffusion of information on mosquito biology and on control strategy, methods and tools; ii) interventions focused on mechanical mass destruction through incineration of eggs laid in large amounts of ovitraps loaded with Bti; and (iii) indoor collections of adults using aspirators, targeting places considered as highly important for virus transmission, such as health units, schools and premises located within hotspots of mosquito density.

For the control activity of mass-trapping eggs, a control-ovitrap (C-ovt) was designed and made of recycled PET bottle washed and paint in black. Bti was applied as a larvicide in 1.5 litter of water per trap. A 40×20 cm cotton fabric, covering the trap’s inner wall, was used as the egg support, according to Lenhart et al. [22], instead of wood paddles. Ten thousand C-ovt were produced at a cost of R$ 0.97 (0.6 US$) each. An adhesive label with information on trap function and a telephone number for contact was put on the house wall near the C-ovt. For security reasons, each municipal government was responsible for supplying Bti-based larvicides, inspecting traps, and incinerating the egg supports.

Each municipal health department designed its own intervention plan according to the local characteristics and resources available. Members of the scientific team provided expertise in entomology.

*SCC:* starting on October 1^st^ 2009, 2 C-ovt per building were installed in 17 health care units (HCU - hospitals, clinics, etc); 1203 traps were distributed, 2 per premise, over hotspot areas of mosquito density located in different neighborhoods; 4473 traps were concentrated in Rio Verde and Santa Tereza, neighborhoods considered as being critical for dengue cases. Overall, around 5680 C-ovt remained installed for two years. As an initiative of the EA team, larvivorous fishes taken from local sources were introduced as larval predators into cisterns, the main local water reservoirs. Fishes popularly known as “piabas”, belonging to a group restrict to South America, the genus *Leporinus* Spix 1829 (Characiformes: Anostomidae), were used. Two to four fishes per cistern were added to 4690 cisterns in January–February/2010 in the neighborhoods of Dona Lica and Palestina, and to 2415 cisterns in the neighborhoods of Cruz Alta, Malaquias, São Miguel, Centro, Nova Sta. Cruz and Bela Vista, in January-February 2011, totaling 7105 treated cisterns. According to the EA, treated sites are inspected at every two months during the regular house visits, and fishes are replaced when mosquito larvae are seen alive. However, no method was employed to evaluate the reduction/elimination of mosquito larvae by this process. *IpojucaMS*: during the first week of October 2009, about 2700 C-ovt were set up, two per household, in the hotspots of the neighborhoods of Centro and Campo do Avião, the latter showing the highest incidence of Dengue cases.

Overall, approximately 8.4 thousands C-ovt were set up in SCC and IpojucaMS. The fabric of each ovitrap used as a support for egg was taken by the EA team at every two months, appropriately incinerated and replaced with new ones. The EA also replaced water and Bti in the traps. Over 25 months, from October 2009 to October 2011, 12 mass-trapping cycles were carried out. Information was offered through a public exhibition, radio, television, banners, posters and leaflets, in order to make the public aware that the fewer available potential breeding sites in a house, the higher the amount of eggs to be laid by mosquitoes in the ovitraps.

Due to the limited availability of field work personnel, mosquito aspirations were not carried out in the scale and frequency expected to potentially contribute for reducing mosquito population size. For collection of adult mosquitoes inside buildings, a light-weight, battery-powered aspirator was used (Horst Armadilhas, SP, Brazil). With the householder’s help, an EA carried out the catching of resting mosquitoes for approximately 20 minutes. Caught mosquitoes were identified to genus/species, counted and destroyed. Aspirations were carried out in SCC (*i*) monthly or fortnightly in 17 Health Care Unities (HCU) throughout 34 months starting on January 2009 (*ii*) and eventually in premises within mosquito density hotspots. Data from aspirations were used for mosquito surveillance. However, in IpojucaMS indoor collections of adult mosquitoes were restricted to a few aspiration sessions when only four *Ae. aegypti* specimens were collected, besides 1236 *Culex quinquefasciatus*.

*Impact of the control interventions*. Baseline data on the presence and density of *Aedes* eggs gathered for 16 months (May/2008 to Sept/2009) before the beginning of the control actions were used to assess changes in population density during the control pressure phase launched in the dry season that lasted from Oct/2009 to Oct/2011 (Table 1). Additionally, data from adult mosquito collections from places considered critical for virus transmission, acquired in the pre-control and control phases, provided additional data on the mosquito population.

**Table 1:** Schematic representation of the study design in both study sites SCC and IpojucaMS, Pernambuco, Brazil.

Activities	2008				2009						2010						2011					
	3	4	5	6	1	2	3	4	5	6	1	2	3	4	5	6	1	2	3	4	5	6
***Mosquito surveillan*** *ce*																						
Sentinel-ovitrap network	**+**	**+**	**+**	**+**	**+**	**+**	**+**	**+**	**+**	**+**	**+**	**+**	**+**	**+**	**+**	**+**	**+**	**+**	**+**	**+**	**+**	**+**
Mosquito aspiration					**+**	**+**	**+**	**+**	**+**	**+**	**+**	**+**	**+**	**+**	**+**	**+**	**+**	**+**	**+**	**+**	**+**	**+**
***Integrated control measures***																						
Mass elimination of eggs									**+**	**+**	**+**	**+**	**+**	**+**	**+**	**+**	**+**	**+**	**+**	**+**	**+**	**+**
Larvivorous fishes*											**+**	**+**	**+**	**+**	**+**	**+**	**+**	**+**	**+**	**+**	**+**	**+**
Education for source reduction									**+**	**+**	**+**	**+**	**+**	**+**	**+**	**+**	**+**	**+**	**+**	**+**	**+**	**+**

* only applied in Santa Cruz do Capibaribe.

It is noteworthy that the entomological routine activities of the official program, PNCD, implemented since 1997– larval search to estimate the entomological indices PI and BI, as well as the use of larvicides - were not interrupted during the study period (2008–2011).

### Data Analysis

A spatial smooth kernel density estimator (KDE) [23] was used to produce surface maps of eggs collected in the S-ovt network, over successive counting cycles, in order to identify hotspots of vector density. Although KDE represents a simple alternative to analyze focal behavioral patterns, it does so by estimating the intensity of the point process throughout the study region. It is based on observed data, from which an unobservable density function thought of as the density according to which a large population is distributed, is estimated. The eggs collected in this case are usually thought of as a random sample from that unknown population. It is also a non-parametric estimation technique, which is good once there is no known parametric model for *Aedes* eggs distribution over urban spaces. And, most importantly, the outputs of the data analysis should be readily readable and easily understood by health staff at all levels (managers, field agents, planners, etc) and by city residents. The Density Maps of *Aedes* eggs produced by KDE procedure fits quite well this need. For the experiments in the SCC and IpojucaMS the KDE parameters were a quadratic function for the KDE kernel and a bandwidth of 300 m and 150 m. This radius covered approximately 12 S-ovt in SCC and 7 S-ovt in IpojucaMS considering each stage of the smoothening phase. Thus, the selected bandwidth allowed the smoothening to be performed with an appropriate number of traps and was chosen based on entomological parameters tested and adjusted over data collected on the local sites. The same grid and color definitions were applied for all cycles, in order to compare results across time. Analyses were performed with TerraView (www.dpi.inpe.br/terraview), an open GIS application.

Due to non-normality and overdispersion distribution of the eggs per ovitrap we have represented these distributions through their mean, minimum and maximum observed. The nonparametric Mann-Whitney test based on ranks was used to compare egg densities (eggs per trap per month). To evaluate the results achieved in reducing the intensity of mosquito eggs, we compared the series representing the number of eggs collected per ovitrap in the twelve mosquito surveys by plotting the median and the 25th and 75th percentiles for each period and each city.

The number of eggs collected per C-ovt was estimated in a sample of 100 oviposition supports (cotton fabric) from SCC and 50 from IpojucaMS, and used for calculating the amount of eggs suppressed at every cycle. According to a method developed by E.V.G. Silva & M.A.V Melo-Santos (unpublished data), the following categories were used for egg number estimation: zero, 1–100, 101–500, 501–1000, >1000. To estimate the total number of eggs suppressed at each mass-trapping cycle, the geometric mean of eggs from the C-ovt sample was calculated taking the mean points of the categories described above, and multiplying the mean by the total number of C-ovt.

## Results

### Operational Features of the Monitoring System

To set up the sentinel network, 32 health agents spent 9 h to install 262 S-ovt in SCC, and 16 agents spent 4 h to install 75 traps in IpojucaMS. Overall, one hour was required for each sentinel-station, including the first visit formalities, trap installation and geographical coordinate record. There was 100% acceptance by the householders. Monthly checking of the traps was carried out in one or, more often, two working days per month by the local EA. Trap maintenance was integrated as a monthly routine activity from May 2008 to October 2011, without significant operational surcharge for the Municipal Dengue Program. However, trap paddles containing eggs were not read every month. At first, due to the large amount of eggs (>400 thousands) collected in the first survey in SCC and IpojucaMS, three full-time technicians spent one month counting eggs on 674 paddles (two per trap) under stereoscopic microscopes, an average of 26 min per paddle. Egg counting was then discontinued until a prototype of the counting support system SDP was set up in the Department of Entomology/Fiocruz-PE in Recife where the paddles were read. Based on egg counting of 6600 paddles using the SDP system, a technician was able to read an average of 6.25 paddles per hour spending approximately 9.6 min per paddle.

### Infestation Intensity before SMCP-*Aedes* Control Actions

**Figure 2:**
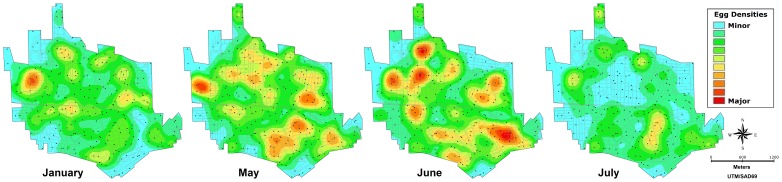
Spatial distribution of *Aedes* eggs in Santa Cruz do Capibaribe-PE, Brazil, in 2009. Each kernel smoothed map of egg densities is based on the number of eggs deposited throughout one month in 262 sentinel-ovitraps placed over a 5.6 km^2^ urban area in Santa Cruz do Capibaribe, at different times along 2009.

The 1^rst^ survey, undertaken in May 2008, showed wide distribution of *Aedes* population over the two territories: in IpojucaMS trap positivity was 92.6% and in SCC it was 100%. Marked differences (p<0.1%) in infestation intensity were however observed amongst sites: densities were 542.9 (0–2207) and 1587.2 (67–6027) eggs/trap/month in IpojucaMS and SCC respectively. Furthermore, the spatial distribution of egg densities revealed evident intra-site heterogeneity and high density clusters, as shown in kernel maps (Figure 1). The subsequent surveys carried out before implementation of the new control strategy, showed the persistence of widespread infestation by *Ae. aegypti* over the study territories, as indicated by 95.8% ovitrap positivity in IpojucaMS in June 2009, slightly higher than that observed one year before (92.6%) but with a lower density: 283.9 (0–1191) eggs/trap/month (p = 0.009). In SCC 100% trap positivity was recorded in the four surveys carried out in 2009 (January, April, June and July). Temporal fluctuations in egg densities showed growing active population densities from January (997.6 (7–5889) eggs/trap/month) to June (1461.1 (6–8209)) (p<0.1%), followed by fast decay in the following month to 762.9 (5–4732) (p<0.1%). The density in June/2009 was similar to that recorded the same month one year before (1587.2 eggs/trap/month). In addition to temporal variations, heterogeneous spatial distribution of densities was observed in each survey (Figure 2).

### *Aedes* Species

*Ae. aepypti* was the only species identified in a sample of 1569 larvae reared from eggs laid in 190 S-ovt in SCC, while in IpojucaMS 2.2% of 1812 specimens reared from eggs deposed in 36 ovitraps were *Ae. albopictus* and the other 97.8% were *Ae. aegypti*.

### Infestation of the Health Care Unities

**Figure 3:**
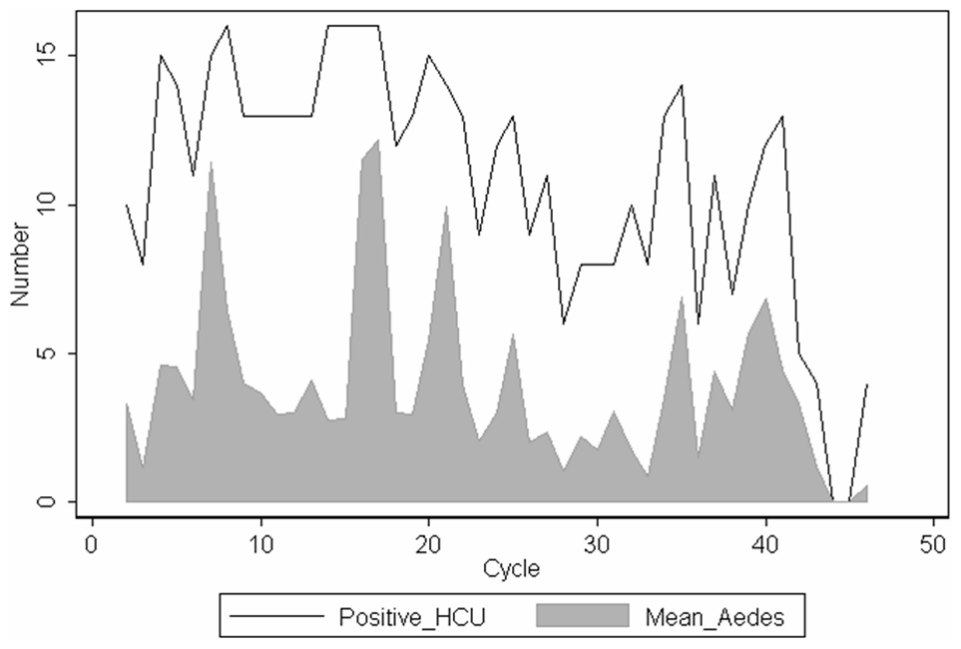
Results of indoor collection of mosquitoes in health care unities in Santa Cruz do Capibaribe-PE. Number of positive sites out of 17 surveyed health care unities (HCU), and mean number of adult *Aedes aegypti* collected through indoor aspirations carried out fortnightly or monthly in HCU in Santa Cruz do Capibaribe-PE, from February 2009 to October 2011.

In SCC, 47 cycles of indoor mosquito aspiration performed in 17 HCU and encompassing 893 events resulted in the collection of 3280 *Ae. aegypti*, of which 62.2% were females, and 9336 *C. quinquefasciatus*. Every HCU has been found to be infested by *Aedes* in several rounds over the study period (Feb/2009 - Oct/2011). More than 10 (11–16) of them were found positive in most rounds along the pre-intervention period and such a high proportion of infested HCU persisted until August 2010 (Figure 3). A lower number of unities (6–11) remained positive in the following rounds from September 2010-May 2011, and none or very few (0–4) HCU were found to be positive for *Aedes* in the last months (June-October 2011). The mean number of *Aedes* per 17 HCU per aspiration round was approximately three to four, rarely reaching more than 10 (Figure 3). It is noteworthy that only in the last six cycles a decreasing trend in mosquito infestation could be observed, indicating the need for a continuous surveillance.

### Control Actions and Social Engagement

The field workers showed proficiency in understanding data on mosquito density distribution expressed through the kernel maps. The health staff, supervisors and field workers continuously demonstrated great interest in discussing the surveys’ results and planning the next actions aiming at further reducing the mosquito population. More than 2000 people in each site visited the public exhibition on the materials related to the proposed control strategy. The activities related to PET bottles collection and the production of C-ovt proved to be a motivating process that involved engaged groups from governmental and non-governmental organizations: health workers of the Dengue and other programs, local school segments - students and schoolteachers, and social organizations as volunteers; moreover, these activities stimulated people to participate in the distribution and set up processes of the traps. The installation of approximately 8000 C-ovt in a few days was in part due to the social engagement.

### Egg Suppression

**Figure 4:**
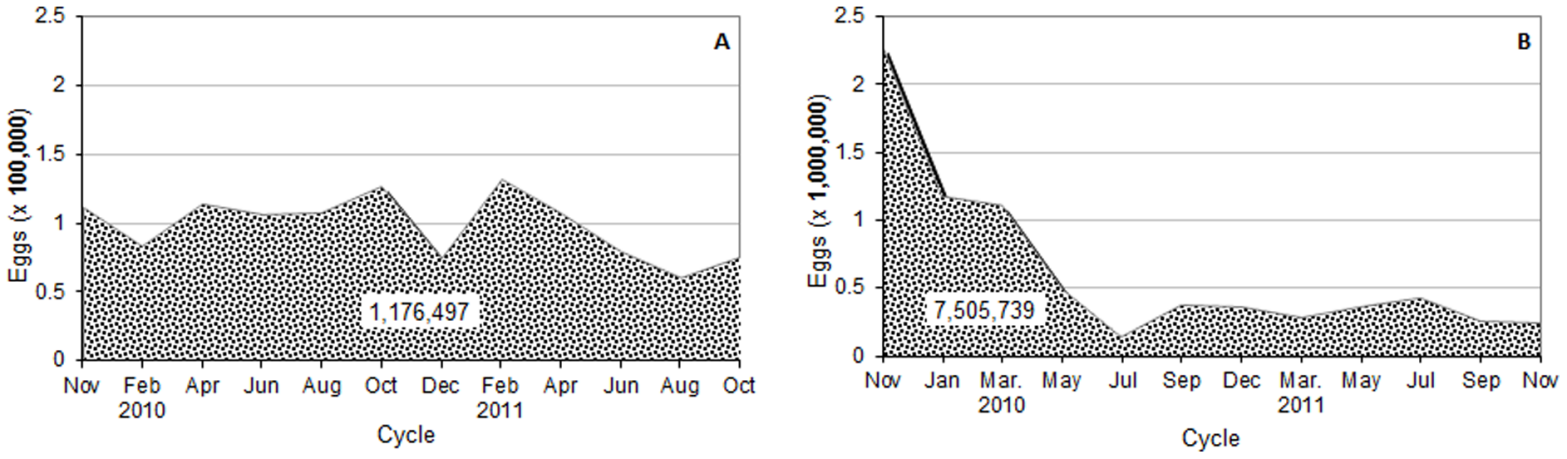
*Aedes* eggs trapped in bimonthly cycles. Estimated amounts of eggs suppressed at every two months through continuous mass-trapping using approximately 2700 control-traps with Bti in the Municipal Seat of Ipojuca (A) and 5700 in Santa Cruz do Capibaribe (B), from October 2009 to October 2011.

The continuous use of thousands C-ovt throughout 26 months starting in the dry season 2009 resulted in the incineration of millions of eggs in both cities. In IpojucaMS, where 2760 C-ovt were concentrated in the neighborhoods of Centro and Campo do Avião, it was estimated that more than 111,000 eggs were incinerated at the end of the 1^st^ bimonthly trapping cycle (Figure 4A). An estimated total of 1.17 million eggs were incinerated over 12 trapping cycles. In SCC the amount of eggs trapped in about 5.7 thousands C-ovt decreased markedly from the 1^st^ (2.26 million eggs) to the 2^nd^ cycle (1.18 million eggs), and starting at the 5^th^ cycle 300–400 thousand eggs were suppressed at every cycle (Figure 4B). A total of 7.5 million eggs were estimated to be incinerated in SCC over the whole period.

### The Impact of Integrated Control Actions on Mosquito Population

**Figure 5:**
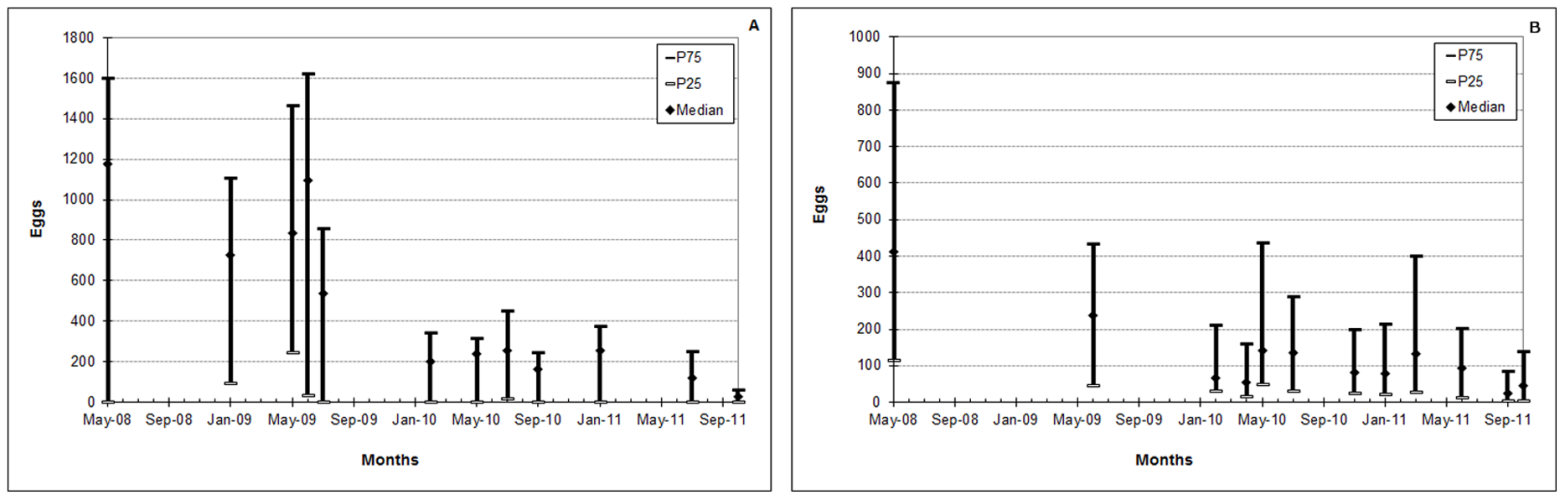
*Aedes* eggs collected in sentinel-ovitraps in Santa Cruz do Capibaribe (A) and Ipojuca MS (B). Median, and 75 and 25 percentiles for the quantitative series of collected eggs before (May/2008 to Sept/2009) and during (Oct/2009 to Oct/2011) the SMCP-*Aedes* control interventions.

**Figure 6:**
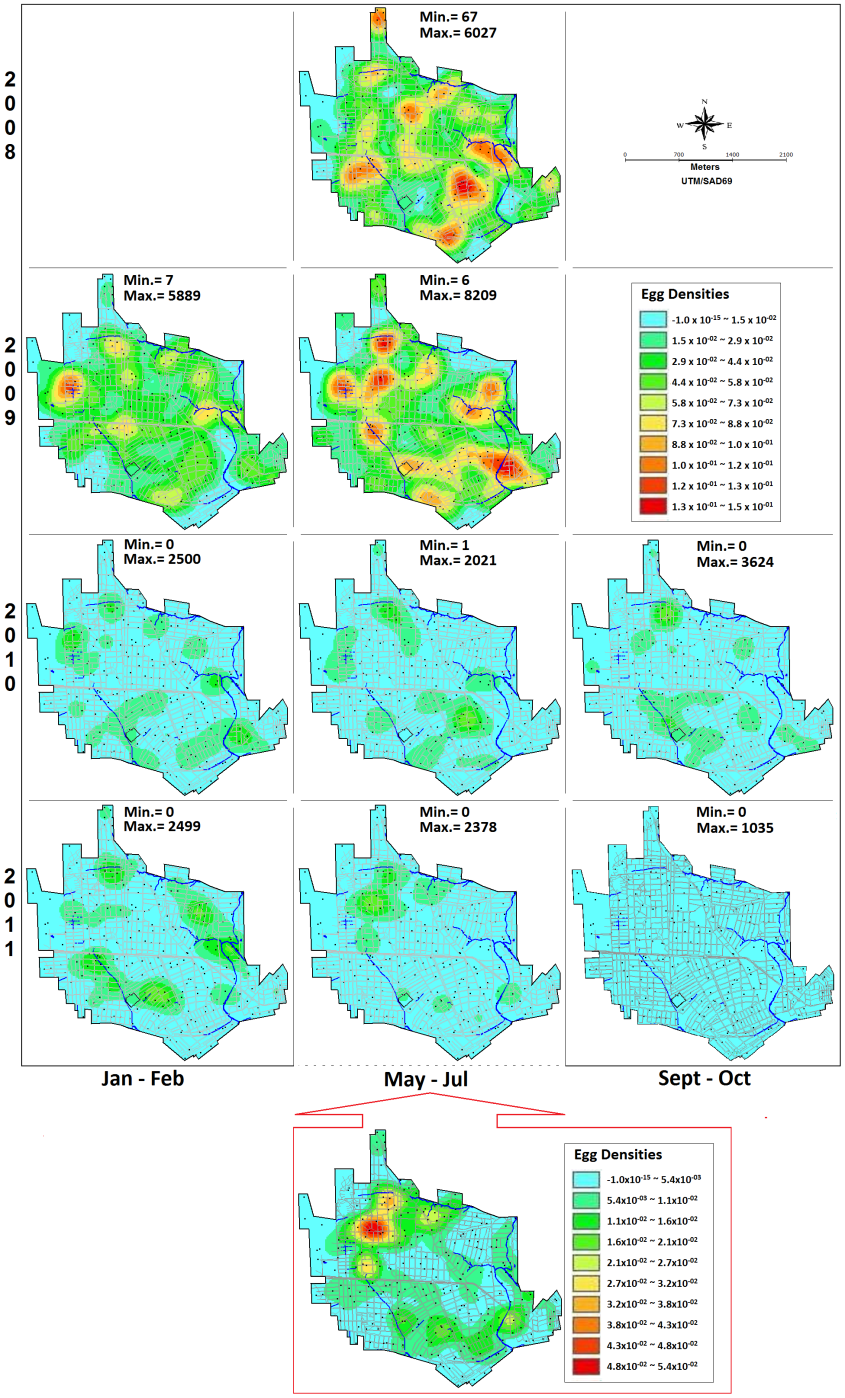
Kernel smoothed egg distribution in Santa Cruz do Capibaribe-PE, Brasil, 2008 to 2011. Maps are based on eggs laid in 262 sentinel-ovitraps during low and high egg density seasons, before (May/2008 to Sept/2009) and during (Oct/2009 to Oct/2011) integrated control measures including mass-suppression of eggs using 5700 control-ovitraps, and larvivorous fishes added to around 7 thousand cisterns. The same scale was used for all compared kernel maps. For each survey, a self-scale kernel map was also constructed to highlight hotspots, as that shown on the bottom of the middle column, for July 2011.

The median and 75 and 25 percentiles of the egg-counting series before and during the control interventions in SCC are shown in Figure 5A, where a marked decline in the median egg density values can be observed. An estimated 90.5% reduction in egg density was observed in 2011 when the median number of eggs in the period of high mosquito abundance (May-July) was compared with that of 2008. Moreover, a relevant drop in the 75 percentile was recorded, where less than 62 eggs were found in 75% of traps in the last counting cycle. The reduction in infestation intensity can also be clearly seen when comparing smoothed kernel maps before and during interventions (Figure 6). Lastly, considering that the main aim of the present study was to serve as a guide for public health activities, self-scale density maps were constructed for each period in order to facilitate the identification of priority areas by the field team (for instance, the map at the bottom of Figure 6).

However, while egg densities decreased progressively during the control intervention period, the trap positivity index remained 100% for at least one year. This index decreased to 98.3% in Jan/2011, to 95% in July and to 82.1% in the last survey, October 2011.

**Figure 7:**
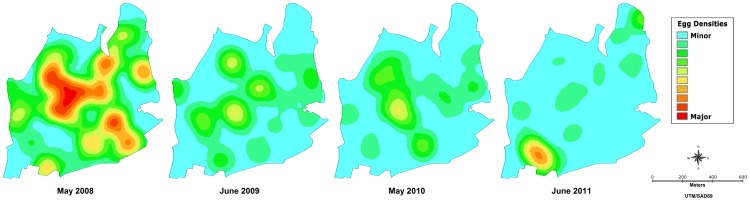
Smoothed spatial distribution of *Aedes* eggs at the high density period (May-June) in Ipojuca, Brazil, 2008–2011. Kernel maps based on the number of eggs deposed in one sentinel-ovitrap during one month. Data from a sentinel network of 75 ovitraps distributed over a 0.8 km^2^ urban area. Mass suppression of eggs using 2700 control-ovitraps started in October 2009.

Data from IpojucaMS also showed important reduction in mosquito population (Figures 5B and 7), with a 77.1% decrease in the median egg density, as estimated comparing June 2011 to May 2008. In fact, the median value dropped from 413 in May 2008 to 94.5 in June 2011 notwithstanding the value of the 75th percentile remained above 130 eggs in the last survey. Data from February/2010 to October/2011, within the control intervention period, reported significantly lower densities than before interventions.

## Discussion

The results of the present study showed that the SMCP-*Aedes*, evaluated at real scale in two different municipalities, was effective for monitoring dengue vectors and that it work as a decision support system. The system effectively indicated, in both localities, a wide spread infestation with moderate to high mosquito densities persisting for more than one year pre-intervention, followed by a progressive and marked decrease in mosquito densities throughout the two years of implementation of integrated control measures.

The infestation scenario of the study areas by *Ae aegypti* found in May 2008 was typical of well-established mosquito populations, as demonstrated by high to moderate amounts of eggs laid in almost all ovitraps, which attests the presence of reproductively active females over the whole territory. Infestation levels remained very high in SCC and IpojucaMS in the following year: 96 and 100% trap positivity. Similar widespread infestation was previously observed in another city of Pernambuco State, when the overall positive ovitrap index remained close to 100% over 5616 trap observations throughout 12 months [7]. More recently, using the same monitoring system we observed an equally widespread infestation by *Ae. aegypti* in Fernando de Noronha Island, an oceanic island 545 km far from Recife (unpublished data).

While the SMCP-*Aedes* showed that *Ae. aegypti* was installed in almost all premises in both study areas, the Premise Index (based on visual detection of larvae) indicated very low infestation levels in the same territories and periods, estimating no more than 3.8 to 12.9% positive houses in SCC and 0.4 to 0.8% in Ipojuca. Such partial site occupation would, if true, characterize recent colonization by the mosquito, a hypothesis incompatible with the annual occurrence of many dengue cases in both cities over the last 12 years. While recent invasions by urban mosquitoes are characterized by limited space occupation, translated into low premise index, a widespread dispersion is conversely considered as an indicator of long-lasting mosquito establishment.

The present results provide a good example of the ability of trap-based quantitative methods on distinguishing different degrees of infestation intensity among sites presenting similar levels of mosquito spatial dispersion: the first mosquito survey showed similar rate of infested houses, while mosquito population size based on egg density was three-fold higher in SCC than in IpojucaMS. Local rainfall characteristics cannot explain the higher density of *Ae. aegypti* in SCC, where the average of annual rainfall was only 360.3 mm, against 1719 mm in Ipojuca. Furthermore, mosquito densities recorded in SCC in 2008–2009 were near as high as those previously observed in Recife [7], with high levels of rainfall (2455 mm per year). This apparent contradiction is possibly due to other factors such as the habits of domestic water storage and spatial distribution of rainfall associated with the terrain conditions, more relevant for *Aedes* establishment and proliferation than rainfall intensity taken as an isolated variable. Another factor possibly contributing for mosquito abundance in SCC was the great number of large cisterns (at least one per house), which in spite of being well covered, were not mosquito-proof. These underground potential breeding sites are the main reason for the large amount of larvicide used in the city. It is possible that an exaggerated confidence on the use of larvicides for controlling mosquito larvae in those habitats account for the maintenance of the high infestation levels observed, since (i) a wide diversity of other types of breeding site are available for mosquito oviposition as is the case in most tropical urban areas, and (ii) *Ae. aegypti* population became resistant to temephos, the organophosphorus larvicide regularly applied mainly in cisterns for more than one decade (Melo-Santos, unpublished data).

The mosquito surveys carried out throughout 2009 indicated that April-June was a period of high mosquito reproduction in SCC. It is reasonable to speculate that the existence of a period with higher mosquito abundance is partly due to other available larval habitats besides the cisterns, as these are located indoors and filled with tap water, being unaffected by rainfall. A few longitudinal studies employing trap-based survey methods to quantify mosquito abundance allowed the assessment of spatial and seasonal patterns of *Ae. aegypti/Ae. albopictus* populations density influenced by climatic factors and by the availability of water-filled objects affected by rainfall [7], [8], [11], [24]–[30].

Investigating possible factors associated to fluctuations in *Ae. aegypti* egg densities along two years in Recife, we observed [31] that although variations in temperature were subtle throughout the year in Pernambuco, egg densities were lower in months of lower temperature, supporting the assumption that temperature possibly also contributed for higher mosquito densities in SCC where annual mean temperature was ∼1°C higher than in Ipojuca.

The spatial distribution of eggs displayed in smoothed maps for both villages showed to be a sensitive indicator of spatial concentration spots of vector reproduction activity (blood-fed active females), thus being able to indicate priority areas for control. This pattern of density hot spots was observed in all areas where the SMCP-*Aedes* was applied - Santa Cruz do Capibaribe, a district of Ipojuca, seven Recife neighborhoods and the Fernando de Noronha Island, showing a clear focal distribution of the *Ae. aegypti* population. This is consistent with a highly clustered spatial pattern observed in several studies on the distribution of *Ae aegypti* carried out in urban areas of different countries and using different sampling methods [32]–[37].

The scale at which *Aedes* reproduction concentrations were evident reinforced the well-known fact that the household environment is a determinant factor for vector breeding activity. This is in agreement with the unique habits of *Ae. aegypti*, a species that seldom moves out from the household with adequate conditions for blood feeding, resting, eggs laying and post-embryonic development [2], [24], [38]. The maps worked as an easy-to-use decision-support tool, as they were easily read and discussed by the health staff including the field workers, helping to decide how and where to direct further control efforts.

The results of this study stress the importance of using an efficient surveillance system, not only to know the real scenario of infestation, but specially to understand its local determinants to define appropriated control strategies.

It is worth mentioning that the scenario of high infestation by the dengue vector, which indicated a mature step of colonization (long-lasting establishment), was observed in areas that have been under systematic bimonthly use of larvicide for fourteen years. While monitoring mosquito population densities for two years in different Brazilian cities subjected to regular temephos-larviciding, as Santa Cruz and Ipojuca (2008–2009) and Recife (2004–2005), no reduction on mosquito population was observed. These findings, together with those performed in other Brazilian cities (Salvador-BA, Manaus-AM, and Rio de Janeiro-RJ) also showing high infestation by *Ae. aegypti* [8], [39], [40] after a long period of regular larviciding, indicate a lack of effectiveness of the classical larvicide-based *Aedes* control strategy applied in Brazil since 1998.

Integrated control actions targeting mosquito eggs and larvae with social participation impacted the mosquito population and caused an overall reduction of around 77% and 90% in egg densities, in the study sites, as compared to the pre-intervention period. This important decrease in population size was only reached at the end of two years of sustained control pressure imposed mainly through continuous eggs trapping, which resulted in more than 8.5 million eggs burned out. In both locations a clear decrease in egg density after the first mass-trapping cycle was efficiently detected by the sentinel ovitrap network in the following month. A sustained gradual reduction was subsequently observed through the sentinel traps. However, dispersion of the mosquito population remained surprisingly unchanged in SCC and slightly oscillated in Ipojuca for at least one year after the beginning of the control actions. Even at the end of two years, more than 80% of the traps remained positive for *Aedes* eggs in both sites, although many of them received just a few eggs per month. These observations clearly indicate that the percentage of positive houses is not a sensitive indicator either for vector population size or for host-vector exposure.

It should be mentioned that the contribution of this form of massive removal of eggs for the success in reducing vector population goes beyond the physical destruction of eggs laid in C-ovt traps. Constructed from plastic bottles the C-ovt embodied a symbolic value in addition to its didactic role and mobilized the community in all stages of the process. This included the active participation of people, mainly students and housewives, (i) in collecting bottles, (ii) in the handmade construction, distribution and installation of traps, (iii) in explaining how they work and persuading householders to adhere and (iv) to visually observe, after each cycle, the eggs deposited in the traps in their houses. With small capital investment, the process contributed to the knowledge of mosquito biology and gave people a real opportunity to work directly in the project with access of visible results and thus to contribute to prevent the proliferation of mosquitoes in their home environment, more so than the large sums spent on publicity in general.

In addition to this “lure and kill” strategy, larvae elimination through predacious fishes added to thousands of cisterns in SCC certainly contributed for reducing mosquito population and, very importantly, prevented the water stored in those cisterns from being contaminated with organophosphorous insecticides.

Although mosquito aspirations inside the houses were not performed at the frequency and length as planned, more than 3200 adult *Ae aegypti* were collected along three years from 17 health facilities in SCC. Considering that public locations could be the main source of DENV spread [41], data on the presence of mosquito females in Health Care Unities, a place where people with DENV infection suspicion proceed to, is of high epidemiological significance. The presence of *Ae. aegypti* in those places was very persistent, with the vector being found in most of the HCU in almost all surveys along the first (preintervention) year of aspirations. Importantly, 100% clearance was observed only at the end of the third year. The results from indoor aspirations: *i)* provided an additional evidence of the high infestation level in SCC, and the sharp reduction in mosquito population after two years of sustained control pressure, as shown by the sentinel-ovitrap network; *ii)* very possibly contributed to limit the number of dengue infection cases; and *iii)* contributed to improve the welfare of patients by suppressing a large number of *Culex* mosquitoes in addition to *Aedes.*

It is known that for *r*-strategist species, like most mosquitoes, a very high proportion of individuals have to be constantly killed in order to impose a strong and sustained control pressure to reduce the target population, as these species are able to recover quickly even after catastrophic mortality [42]. The ways to reach the required control pressure seems to be quite different for the main urban disease vectors *Culex* and *Aedes*. For the lymphatic filariais vector *Cx. quinquefasciatus* (Say) which lay grouped eggs in well-defined sites and use an oviposition aggregation pheromone, continuous pressure can be obtained through larval control, as shown in field trials [43]–[47]. Differently, for the dengue vector *Ae aegypti*, its unique oviposition habits result in a wide spread of its progeny in a very large amount of several breeding sites, and the integration of methods directed against at least two mosquito developmental stages seems to be essential for an effective vector control. In spite of enormous efforts to control the dengue vectors in recent decades, very few successful real-scale experiences have been reported in the scientific literature. Rare examples are the elimination of dengue transmission through community-based biological control of the vector in Vietnamese villages [48], [49]; integrated control actions including sticky and lethal ovitraps, source reduction and residual spraying directed at dengue cases on Thursday Island/Australia [15]; and the integration of source reduction, copepods, Bti and lethal ovitraps in rural villages in Thailand [17]. Social engagement and the use of control methods targeting more than one mosquito developmental stage are the main features of these examples.

We evaluate that the success in markedly reducing *Ae. aegypti* populations in SCC and IpojucaMS can be attributed to the appropriate choice of control measures for sustained mass elimination guided by a sensitive and easy-to-use surveillance system, which was capable to motivate the health staff. Despite a much higher infestation degree in the pre-intervention period, the impact of interventions on mosquito population was greater in SCC. Although it is difficult to measure the contribution of people’s involvement to the program’s success, one factor seemed evident: the municipal authorities and health managers demonstrated to be more intensely involved and thus to create a greater commitment of the health staff in SCC than in Ipojuca. One of the factors possibly contributing to the greater commitment of the SCC population was that the actions covered an urban area where over 97% of the municipal inhabitants live, improving the notion of their help in reducing dengue transmission. As a consequence, control measures were more intense and residents possibly received more stimuli for positive actions to avoid mosquito breeding. Furthermore, social segments such as school communities participated more actively in control actions in SCC.

In practical terms, the observed reduction in egg density can be expressed as a decrease in the number of *Ae aegypti* active females ovipositing over a thousand eggs per month in each house, as observed in SCC, to only one or few females laying approximately one hundred eggs per month in 80% of the houses. The association of DENV transmission with the abundance of *Ae aegypti* has been demonstrated [50], however the threshold of vector density under which no virus transmission would occur is yet to be defined. This threshold is supposedly very low because by feeding preferentially and frequently on human blood *Ae. aegypti* exponentially boosts the basic reproduction rate of virus transition [2]. Historical series on dengue incidence comparing SCC and other cities within the same geographical region in Pernambuco are being analyzed in order to investigate whether the marked decrease in vector population size has any impact on DENV transmission.

Several trap models have been evaluated in regards to their potential to be used in large scale strategies to promote mass destruction of adult mosquitoes or eggs, and the development of promising genetic vector control techniques [51]–[54] enhances the options of appropriate approaches for an integrated management of dengue vector populations. In our opinion, the mass elimination of *Aedes* eggs should be considered as an efficient option because it may prevent mosquito population boosts resulting from sudden hatching of large amounts of eggs that have been maintained viable in the environment during dry season [7], and because ovitraps handling is simple and their use favor social engagement to the control program. Furthermore, by mass destructing *Aedes* eggs from endemic areas, DENV viruses present in the eggs may also be eliminated. Although it has not been experimentally demonstrated, it is possible that besides being the biological vectors of DENV, *Ae. aegypti* and *Ae. albopictus* could also play a role as the reservoirs of dengue viruses in urban environments resulting in the persistence of the virus in the environment between periodic transmission periods through infected eggs.
